# Hyaluronic Acid-Decorated Chitosan Nanoparticles for CD44-Targeted Delivery of Everolimus

**DOI:** 10.3390/ijms19082310

**Published:** 2018-08-07

**Authors:** Enrica Chiesa, Rossella Dorati, Bice Conti, Tiziana Modena, Emanuela Cova, Federica Meloni, Ida Genta

**Affiliations:** 1 Department of Drug Sciences, University of Pavia, Viale Taramelli 12, 27100 Pavia, Italy; enrica.chiesa01@universitadipavia.it (E.C.); rossella.dorati@unipv.it (R.D.); bice.conti@unipv.it (B.C.); tiziana.modena@unipv.it (T.M.); 2 Clinica di Malattie del Apparato Respiratorio, IRCCS Fondazione S. Matteo, via Golgi 19, 27100 Pavia, Italy; E.Cova@smatteo.pv.it (E.C.); F.Meloni@smatteo.pv.it (F.M.); 3 Department of Molecular Medicine, Pneumology Unit, University of Pavia, Viale Golgi, 19, 27100 Pavia, Italy

**Keywords:** hyaluronic acid, everolimus, polysaccharides nanoparticles, ionotropic gelification, CD44-targeting, bronchiolitis obliterans syndrome

## Abstract

Bronchiolitis obliterans syndrome (BOS), caused by lung allograft-derived mesenchymal cells’ abnormal proliferation and extracellular matrix deposition, is the main cause of lung allograft rejection. In this study, a mild one-step ionotropic gelation method was set up to nanoencapsulate the everolimus, a key molecule in allograft organ rejection prevention, into hyaluronic acid-decorated chitosan-based nanoparticles. Rationale was the selective delivery of everolimus into lung allograft-derived mesenchymal cells; these cells are characterized by the CD44-overexpressing feature, and hyaluronic acid has proven to be a natural selective CD44-targeting moiety. The optimal process conditions were established by a design of experiment approach (full factorial design) aiming at the control of the nanoparticle size (≤200 nm), minimizing the size polydispersity (PDI 0.171 ± 0.04), and at the negative ζ potential maximization (−30.9 mV). The everolimus was successfully loaded into hyaluronic acid-decorated chitosan-based nanoparticles (95.94 ± 13.68 μg/100 mg nanoparticles) and in vitro released in 24 h. The hyaluronic acid decoration on the nanoparticles provided targetability to CD44-overexpressing mesenchymal cells isolated from bronchoalveolar lavage of BOS-affected patients. The mesenchymal cells’ growth tests along with the nanoparticles uptake studies, at 37 °C and 4 °C, respectively, demonstrated a clear improvement of everolimus inhibitory activity when it is encapsulated in hyaluronic acid-decorated chitosan-based nanoparticles, ascribable to their active uptake mechanism.

## 1. Introduction

Everolimus (EVE), a macrocyclic lactone derivative of Sirolimus, has recently emerged as a key maintenance immunosuppressive molecule in therapies for preventing acute and chronic allograft organ rejection [1,2,3]. EVE is successfully used in combination with cyclosporine and corticosteroids both in adult and paediatric population of renal- and cardiac-transplant recipients, but far less often for lung-transplant recipients [4,5,6,7]. Particularly for these patients, survival continues to be challenged by chronic allograft dysfunctions, such as obliterative bronchiolitis or its clinical correlate bronchiolitis obliterans syndrome (BOS), a fibrous obliteration of small airways caused by mesenchymal cells (MSc) abnormal proliferation, and extracellular matrix deposition causing bronchiolar occlusion and organ rejection [8,9,10,11]. Although the BOS etiology and pathogenesis are not yet completely known, it is possible to assume the involvement of both alloimmune and non-alloimmune mechanisms [12,13], and the BOS management strategies frequently involve ineffective triple or quadruple maintenance immunosuppressive therapies (i.e., cyclosporine A, azathioprine, and methylprednisolone co-administration) [1,8,12]. Promising results were recently reported, including EVE in clinical trials, showing a significant reduction in the airway obliteration [11]; however, severe dose-dependent EVE side effects have been highlighted during the clinical trials. These side-effects have been ascribed to the narrow therapeutic window of the drug [14,15], ranging in a concentration rank of 3–8 ng/mL [1]. Dose-finding studies suggested that an EVE concentration below 3 ng/mL increases the risk of acute rejections, while the drug concentration higher than 8 ng/mL are associated to toxicity onset, including stomatitis, fatigue, rash, hyperglycemia, hyperlipidemia, and myelosuppression [16]. The unsatisfactory clinical response could be partially due to the EVE systemic toxicity and its insufficient accumulation at the target tissues [14,17].

Therefore, the EVE encapsulation into selective submicron carriers intended for pulmonary administration [18,19,20] represents a viable strategy to specifically target the MSc and reduce their proliferation. Moreover, this approach allows for ensuring adequate EVE efficacy, maintaining a good local tolerability and low systemic toxicity. This challenging strategy could be pursued by exploiting CD44-overexpressing feature of human lung allograft-derived MSc as target for CD44-targeted EVE loaded nanostructures. To the best of our knowledge, only the EVE loaded anti-human CD44-engineered gold nanoparticles (NPs) were synthetized, and their effectiveness in reducing the MSc proliferation was demonstrated by ex vivo cellular tests and by inhalation in normal mice [17,21]. However, despite these encouraging preliminary results, the gold NPs are inert but non-biodegradable carriers and are unsuitable for a long-term therapy.

On that basis, a more friendly and safe CD44-targeted nanocarrier for the selective delivery of EVE is still a real clinical need. Our attention was focused on hyaluronic acid (HA), a naturally occurring glycosaminoglycan polysaccharide composed of *N*-acetyl-d-glucosamine and G-glucuronic acid, with good biocompatibility, biodegradability, no-toxicity, and well-known second generation mucoadhesion property [22]. The targeted interaction of HA with CD44 was widely studied and it is known as a good tool in nanoscale drug delivery to improve nanocarrier cell internalization using the CD44 receptor as anchor of attachment for the physically or chemically HA-decorated nanocarrier, such as liposomes [23,24,25] and polymeric NPs [26,27,28,29,30,31].

Different types of nano-scale HA-based drug delivery systems can be synthetized following different strategies (i.e., HA-drug conjugate synthesis, HA grafted copolymer synthesis, HA coating of various polymeric nanocarriers, HA-based soft nanogel carriers made by hydrophobic association, chemically cross-linking, or ionotropic gelation (IG)) [22,26,31,32,33,34,35]. IG represents the gold-standard method to tailor HA-based nanoscale drug delivery systems [22], because of the mild process conditions and the wide range of cationic polymers proper for HA interacting. Among the latter, chitosan (CS), a linear random copolymer of β-1,4-d-glucose-2-amine and *N*-acetyl-d-glucose-2-amine, derived from deacetylation of chitin [36,37], was largely studied as an additional polymer for the formation of HA-decorated NPs using the IG method [26,38]. CS is a non-toxic, biocompatible, biodegradable polycationic polysaccharide that has excellent mucoadhesive strength and is routinely explored in a wide range of pharmaceutical applications, in drug delivery for micro- and nano-particle manufacturing [35,36,37,38,39,40,41]. Furthermore, the deposition of HA preferentially on the CS-based NPs’ surface can improve in vivo the stability controlling the NPs’ aggregation phenomena [42]. In the conventional IG method, the CS solution is added to a solution containing HA and a proper CS cross-linking agent, such as pentasodium tripolyphosphate (TPP), extensively used for its non-toxic and multivalent properties [43]. In these experimental conditions, CS and HA ionically interact, while TPP triggers a CS liquid-to-gel transition, spontaneously forming NPs, characterized by a neutral or positive Zeta (ζ) potential, indicating that HA is mostly placed into the NPs and that its carboxylic groups are totally neutralized by CS ammine groups [22,33,34,38,44,45]. In order to obtain NPs with a high HA deposition on the NPs surface, further efforts are required to maximize the conventional IG method.

The main goal of the present work was to set up a mild, one-step IG method, named the “inverse” IG (IIG), to prepare the CS-based nanoparticles displaying HA on the surface, and so-called HA-decorated CS based NPs (HA/CS-TPP NPs), providing a biocompatible and biodegradable carrier suitable for selective bronchial MSc targetability and payload delivery, opening up new perspectives for a safe pulmonary therapy for BOS.

In the first step, the optimal process conditions for the synthesis of the HA/CS-TPP NPs suitable for the pulmonary application were identified, as being in the size of ≤200 nm, with a narrow size distribution, and a negative ζ potential. A size of ≤200 nm was chosen as a trade-off between the ability of the NPs to penetrate through the pulmonary mucus after local administration and the chance for interacting with cells; the narrow size distribution should prevent the penetration of NPs into systemic circulation, reducing the adverse side effects and affecting the pulmonary drug efficacy [46,47]; while the negative ζ potential provided evidence for the HA chains coating on the NPs surface. Following this, the EVE entrapment efficiency and in vitro release profile were evaluated for EVE loaded HA/CS-TPP NPs. Ex vivo studies on normal human dermal fibroblasts (NHDFs) and MSc isolated from human bronchoalveolar lavage (BAL) by BOS-affected patients were subsequently performed to estimate the cytocompatibility of HA/CS-TPP NPs and the effect of EVE released from the NPs on the cells proliferation. Furthermore, the CD44-mediated HA/CS-TPP NPs cellular internalization was assessed by the uptake tests performed at 37 °C and 4 °C.

## 2. Results and Discussion

### 2.1. Nanoparticles Preparation Method Set Up

To date, two traditional approaches have been used to obtain HA/CS-TPP NPs, HA coating on pre-formed CS-TPP NPs [48], or conventional IG methods setting among the two oppositely charged polymers, HA and CS, and a CS cross-linking agent (i.e., TPP). The interaction mechanisms between CS and TPP, and between the CS and HA involved in the NPs formation are well-established [34,48,49,50,51].

The ionic gelation technique for the formation of CS-TPP NPs is based on the inter- and intra-molecular ionic linkages created between the negatively charged groups of the TPP, a small ion cross-linking agent with a triple negative charge, and the positively charged primary amino groups of CS [49]. Among the other critical formulation (CS molecular weight and degree of deacetylation, pH of the CS solution) and process (temperature of cross-linking) variables, the CS and TPP concentrations, CS/TPP weight ratio, and volume ratio between CS and TPP solutions are responsible for the success of the gelation process [48,52]. In order to obtain HA/CS-TPP NPs, an excess of CS is necessary to provide positively charged NPs to be suitably coated with negatively charged HA. The HA coating steps on the positively charged CS-TPP NPs result in agglomeration and possibly flocculation, either because of the interactions between the positively and negatively charged patches on the different NPs, or because of the absence of electrostatic stabilization during the intermediate states of the adsorption [48]; the critical step in the surface adsorption of a polyelectrolyte is the inversion of the NPs’ zeta potential, as agglomeration is likely to occur around the isoelectric point [26]. The outcome depends on the concentration and size of both the CS-TPP NPs and HA chains as well as on the strength of their interactions. In general, a multi-steps process finalized to optimize the HA decoration is time-consuming and leads to non-reproducible NPs batches [33,44].

On the other hand, the traditional IG approach among HA, CS, and TPP combines electrostatic interactions between both oppositely charged polysaccharides, CS, and HA, with the concomitant ability of CS to undergo a liquid–gel transition, due to its ionic interaction with TPP. Moreover, HA and CS were expected to interact through the hydrogen bonds and other intermolecular forces [34,45,49]. Conventional IG methods permitted HA/CS-TPP NPs to be easily prepared with a neutral/positive surface charge, these charge values foreshadow an interconnected polymeric structure characterized by a low outer HA decoration [34].

In both approaches, the polysaccharides and TPP concentrations have been shown to affect the NPs’ features, such as size distribution and surface charge; these are considered as the most important parameters for predicting the NPs’ biopharmaceutical behaviour, such as bioavailability, drug release rate, toxicity, and biodistribution [20,53].

In the present study, a mild, one-step IG method was developed for preparing HA/CS-TPP NPs characterized by a high HA surface deposition, for more selective CD44 targeting. Starting from the literature data and exploiting the interaction mechanisms among HA, CS, and TPP, discussed above, we tested an IG method in which a fixed volume of a CS solution was added dropwise to an equal volume of an aqueous solution containing both HA and TPP. TPP has only an ancillary role, because the amount used is significantly lower than the CS and HA themselves. The method was defined as inverse ionotropic gelation (IIG), because the dropping procedure and resulting NPs’ surface charge were inverted with respect to the most conventional IG methods [33,44].

Based on the literature data and our previous studies [44,48,49,54,55,56], a set of preliminary experiments were carried out varying one factor at a time, such as HA solution concentration (0.5–1.0–1.25–2 mg/mL) and CS solution concentration (0.1125–0.3–0.5–0.75–1 mg/mL). The TPP solution concentration was fixed at 0.5 mg/mL to roughly define the approximate ranges for the above-mentioned factors; such experimental conditions allow for the NPs formation, avoiding macroscopic aggregates formation, and the setting of a formulation design space for the following factors.

A screening design of experiment (DoE) was then performed in order to characterize and establish the formulation of the design space, by identifying the parameters (such as HA and CS concentration, and TPP concentration) and their interactions that were found to be crucial in order to define the final product characteristics. With the aim of getting the NPs’ size below 200 nm for improving the cellular uptake and negative ζ potential, as stated in literature, the first proof of the NPs’ surface coating by HA polymer, particles size, and ζ potential results were combined and analysed through multivariate analyses.

The 2^3^ full factorial design was used for investigating the effect of the HA, CS, and TPP concentration on the NPs’ size and ζ potential (see Section 3.2); CS and TPP were used at a high dilution in the final volume (0.015–0.025% and 0.00016–0.00031%, respectively), and HA was used at a relatively low concentration (0.05–0.1%). The process conditions (input variables) tested in the screening DoE, and the relative output variables in terms of the NPs’ mean sizes, polydispersity index (PDI), and ζ potentials, are summarized in Table 1. The data highlighted the NPs’ mean sizes ranging from about 630 to 181 nm, with the low PDI index (0.171–0.421) and negative ζ potentials (−1.1/−40.5) obtained in all of the process conditions.

As far as the NPs’ mean size is concerned, the goodness of fit is 90.40% (R^2^) and it is mathematically stated by the following equation:

Mean size = 295.14 + 81.425 × [HA] − 74.6 × [CS] − 85.725 × [TPP] + − 7.425 × [HA] × [CS] + 35.275 × [CS] × [TPP](1)

where [HA], [CS], and [TPP] represent the HA, CS, and TPP solution concentrations used to prepare the NPs batches.

**Figure 1:**
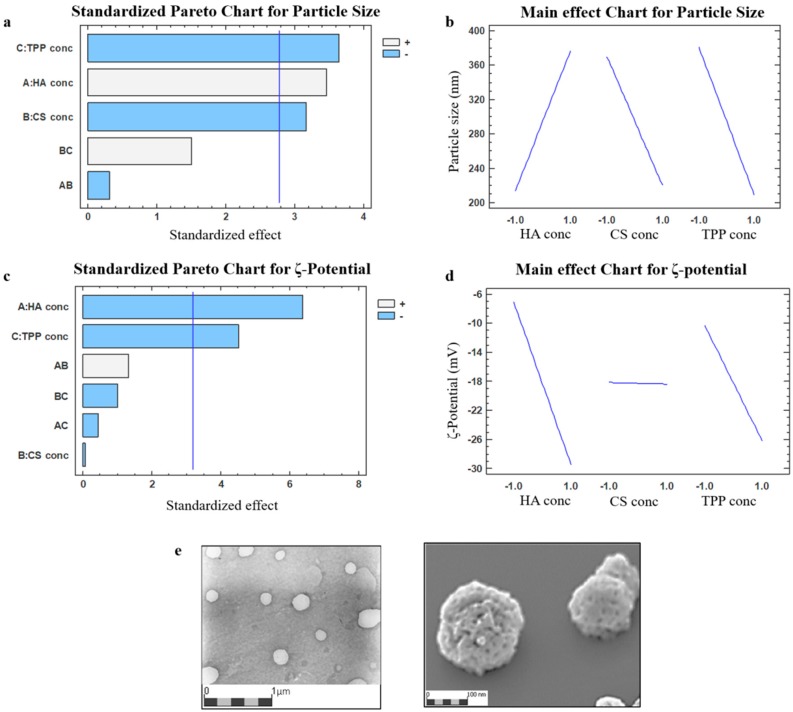
Pareto chart and main effect chart depicting the impacts by individual factors or two-factors interaction on nanoparticles (NPs) size (**a**,**b**, respectively) and ζ potential (**c**,**d**, respectively) in the two-level full factorial screening; (**e**) TEM (transmission electron microscope) and SEM (scanning electron microscope) photomicrographs of the hyaluronic acid (HA)/chitosan (CS)-pentasodium tripolyphosphate (TPP) NPs (DoE batch #B).

The results were graphically represented through a standardized Pareto chart and the main effect chart (Figure 1a,b).

The Pareto chart for particle sizes (Figure 1a) confirmed that the NPs’ sizes can be modulated by varying the polymers’ concentration employed; all of the inputs selected (A: HA, B: CS, and C: TPP solution ratio) significantly affect the particle size with a probability higher than 95% (*p* value < 0.05); while the interaction between the main factors (AB and BC) resulted as non-significant. The HA solution concentration’s main factor had a positive regression coefficient, indicating that a high mean size was obtained when a higher HA concentration was used, as shown in Figure 1b and verified in Table 1 (DoE batches #E/F/G/H, mean size ranging from 242.5 to 630.5 nm). The CS and TPP solution concentrations exerted significant negative main effects on the particle sizes, reducing the NPs’ mean diameter (Table 1, DoE batches#A/B/C/D, mean size ranging from 181.9 to 385.2 nm), because of the more effective interaction between CS and its cross-linking agent.

Theoretically, a high TPP concentration is needed to lead a smaller particle size, because a more compacted nanostructure could be provided by strongly electrostatic interactions [38,55,57]. In the attempt to understand the crucial role of TPP on the NPs’ sizes, both polysaccharides are mixed without TPP, allowing for the formation of heterogeneous and unstable particles (mean size 1607.8 ± 1114.1 nm, PDI 0.479).

The NPs’ formation was triggered by the randomized electrostatic interaction between HA and CS; the supplement of TPP was needed to achieve a more-organized structure in the nanometer range, indicating that the ionic gelation provided by the interaction between CS and TPP is still required for the suitable NPs’ formation. Nevertheless, in our study, extremely high CS/TPP weight ratios (equal to 320:1, 160:1, 36:1, to 18:1, *w*/*w*) were tested. In the literature, high CS/TPP weight ratios were correlated to a slower kinetic of NPs formation [48,52]. This may mean a more controlled process of formation and, at the same time, the ability to promote CS/HA chains’ entanglement and ionic interactions to stabilize the nanostructures.

The PDI values ranging from about 0.1 to 0.4 were considered acceptable and they were indicative of an adequate narrow size distribution for the polysaccharides-based NPs [53]. Satisfactory PDI values (around 0.2, Table 1) were obtained for the DoE batches #A, B, D, G, and L, while the broadest size distribution was detected for the DoE batch #C (PDI = 0.421 ± 0.163, Table 1).

Data regarding the ζ potentials, reported in Table 1, were fitted in the first order polynomial function, resulting in the below mentioned mathematical equation:

ζ potential = −18.27 − 11.195 × [HA] − 0.1225 × [CS] − 7.935 × [TPP] + 2.3175 × [HA] × [CS] + −0.765 × [HA] × [TPP] − 1.7475 × [CS] × [TPP](2)

where [HA], [CS], and [TPP] represent HA, CS, and TPP solution concentrations, the main factors selected for the study.

The value of the determination coefficient (R^2^) is equal to 95.53% for the mathematical model, indicating a good fit of the linear equation. A standardized Pareto chart depicting the impacts of individual main factors as well as a two-factor interaction on the surface charge (Figure 1c), revealed the negative significant influence of the HA (A) and TPP (C) solution concentration (*p* < 0.05). The impact of the CS solution concentration (B) was negligible, as visualized from Figure 1d, this could be attributed to the high HA/CS weight ratios (ranging from 2:1 to 6.66:1) selected for the study [28,33,58].

The set-up one-step IIG preparation method provided the HA/CS-TPP NPs with optimal physical characteristics (DoE batch #B in Table 1), by adding dropwise 4 mL of a CS aqueous solution (0.5 mg/mL), at constant flow rate of 1 mL/min, through 27 G needle into a mixture made of 4 mL of HA aqueous solution at 1 mg/mL (CS/HA ratio 1:2 *w*/*w*) and 50 µL TPP solution at 0.5 mg/mL under magnetic stirring (700 rpm) for 10 min at room temperature. The dynamic light scattering (DLS) analysis revealed the mean size of 181.9 ± 65.6 nm with a PDI value 0.171 ± 0.04. A negative ζ potential around −30 mV indicates that the NPs’ surface is preferably composed by HA, which is crucial for CD44 targeting and the subsequent cellular uptake. Furthermore, the negative ζ potential increases the NPs’ stability in the aqueous media [59].

The yield of the process, defined as the amount of polymer that effectively reacts to form NPs, was around 50% (3.026 ± 0.151 mg/batch); the value is expressed as a mean of three batches.

### 2.2. Nanoparticles Characterization

A morphological examination of HA/CS-TPP NPs was carried out by transmission electron microscopy (TEM), and a representative TEM image of the DoE batch #B (Figure 1e) shows mostly spherical shaped NPs. Image elaboration by Jmicrovision v1.27 revealed a HA/CS-TPP NPs size (210.90 ± 51.01 nm), consistent with the DLS results. The SEM image confirmed the round shaped particles with a rough surface.

The NPs’ polymer composition was assessed as the percentages of HA and CS reacting effectively during the NPs’ formation process. The HA quantification was performed by analyzing HPLC-UV, the supernatants recovered after centrifugation. The HA reacted during the NPs’ formation, and was expressed as a mean of four batches, and the mean value was 1.27 ± 0.32; considering the amount of HA used for each batch preparation (4 mg), the percentage of polymer that effectively reacted with the CS amino groups was 31.7 ± 6.8%. The final percentage of HA was calculated, taking into consideration the processes yield after freeze-drying (about 3.026 mg), and it was 41.97%.

The amount of CS that reacted during the NPs’ formation was determined using Cibacron Brilliant 3B-A and was expressed as a mean of four batches. The value obtained by the colorimetric method was 1.935 ± 0.23 mg, corresponding to 96.8% of the CS initially used (2 mg) for each batch; the final percentage of CS contained into the NPs was 63.93% of CS.

**Figure 2:**
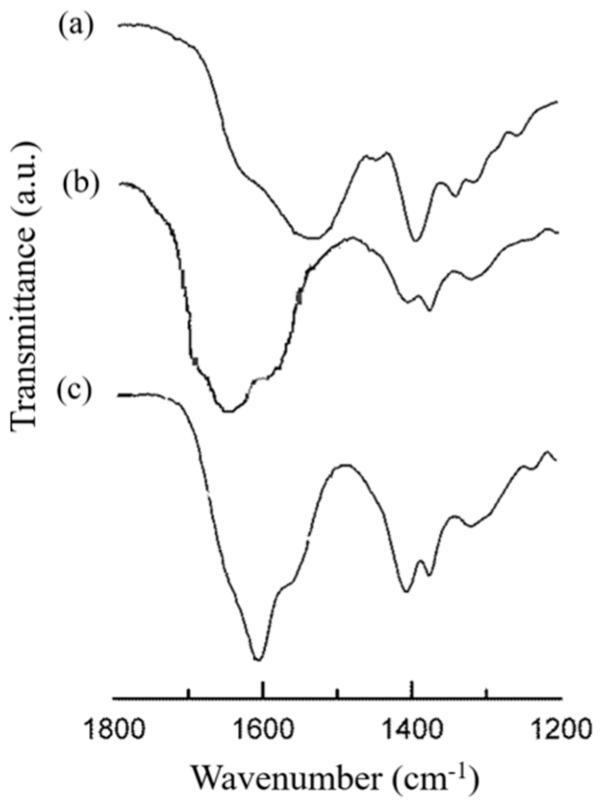
FTIR spectra of (a) CS (raw material), (b) HA/CS-TPP NPs, and (c) HA (raw material).

The identification of both the HA and CS components as well as their interaction during the NPs’ formation was further evaluated by FTIR spectroscopy. The FTIR spectrum of the HA/CS-TPP NPs’ was compared with those of the raw polymers at their fingerprint region (1200–1800 cm^−1^) (Figure 2).

The HA spectrum showed characteristic amide bands of the sodium form at about 1608, 1568, 1407, and 1322 cm^−1^, corresponding to C=O bond, amide II, C–O bond of –COONa group, and amide III, respectively [34,60].

The CS hydrochloride spectrum displays two strong vibrations at 1630 and 1540 cm^−1^, which have been previously attributed to the C=O stretching vibration of amide I as well as to the N–H stretching vibration of the amino group, respectively [61].

The spectrum of HA/CS-TPP NPs presented several characteristic HA and CS vibrations, and shifted to higher wavenumbers. The signal displacement confirmed the involvement of both of the macromolecular chains in the NPs’ formation. A new small shoulder arising at 1730 cm^−1^ is evidence of the HA protonation occurring during the formation of the polyelectrolyte complex, as previously identified [34,62].

### 2.3. EVE Loaded HA/CS-TPP Nanoparticles Preparation

Our first goal was to easily prepare the biocompatible and biodegradable nanocarriers targeting the CD44 receptor, and releasing the EVE in a tuned manner. The EVE loaded HA/CS-TPP NPs were prepared by the set-up IIG method, by adding 200 μL of EVE stock solution (0.1 mg/mL in a mixture methanol:water (1:1, *v*/*v*) to the CS solution, to be dropped through a 27 G needle into a HA/TPP solution, as described above (see Section 3.4).

The EVE loaded HA/CS-TPP NPs were characterized according to the size distribution and ζ potential; the hydrodynamic size, measured by DLS, was 160.2 ± 68.1 nm with a PDI value 0.181 ± 0.02; the comparable size distribution values were noted for the placebo HA/CS-TPP NPs (DoE batch #B, Table 1). The placebo and EVE loaded formulations were in the appropriate size range and homogeneous size distribution. The ζ potential was negative (−31.77 mV), accordingly to the HA surface decoration. The EVE incorporation into HA/CS-TPP NPs affected neither the overall physical properties nor the NPs’ morphology, as visualized by TEM, and the data are consistent with the low drug:polymer weight ratio (approximately 1:100), as discussed below.

The EVE loaded HA/CS-TPP NPs showed a satisfactory drug loading of 95.94 ± 13.68 μg EVE/100 mg NPs; considering the minimum effective EVE concentration reported in the literature [8], the high EVE drug loading obtained by the IIG method should trigger a complete inhibition of the cells’ proliferation.

**Figure 3:**
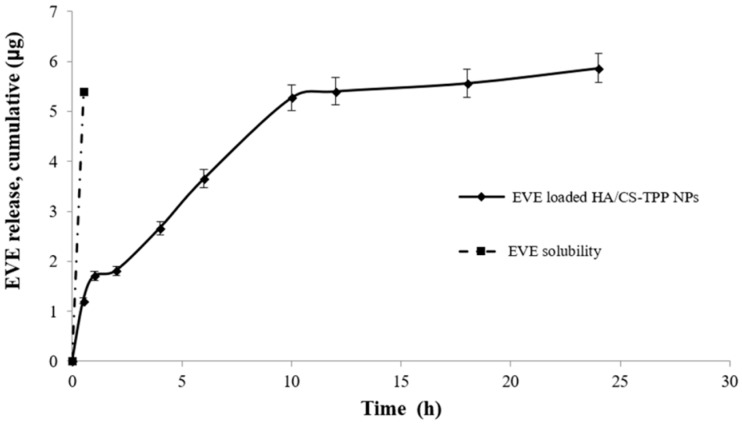
Everolimus (EVE) release profile of EVE loaded HA/CS-TPP NPs in phosphate saline buffer (PBS), at pH 7.4 (supplemented with 0.4% Tween 20).

Figure 3 shows the EVE release profile from the EVE loaded HA/CS-TPP NPs (solid black line); the profile is compared with the dissolution profile of the EVE (raw material) in the media at an equal concentration (dashed line). The EVE dissolution was completed within 30 min.

The EVE loaded HA/CS-TPP NPs shows a prolonged release profile, indicating the ability of the set-up preparation method to effectively encapsulate the EVE drug, having hydrophobic features inside the HA/CS-TPP NPs’ hydrophilic carriers. As previously observed by Janes et al. [63], drug release is governed by the degradation of the HA/CS-TPP NPs’ hydrophilic carrier, which depends on the intensity of the interaction between the polymers and the medium ionic strength. The release profile shows a burst release of about 30% in the first hour of incubation, as a consequential effect of the rapid surface desorption of the drug from a large surface area, provided by the nanoscale particles [64]. The burst release was followed by a slow release, reaching 62% at the sixth h, and the EVE was completely released after 24 h of incubation; the EVE release is controlled by its diffusion through the swollen matrix and by polymer erosion; this last mechanism is prominent in case of hydrogel [36].

The NPs’ swelling phenomena was evaluated by tracking the particle size increase during the incubation at 37 °C in pH 7.4 PBS (supplemented with 0.4% *w/v* Tween 20). A slight NPs size increase (26.05 ± 5.87%) was observed after 1 h, and the HA/CS-TPP NPs presented a mean diameter of 202.50 ± 4.45 nm, with PDI of 0.282 ± 0.012. Subsequently, a noticeable increased particle size was detected after 6 h, and the HA/CS-TPP NPs showed a mean diameter of 792.40 ± 22.63, four-fold higher than diameter at time zero. Moreover, the broadest size distribution was revealed at the sixth h, with a PDI value of 0.608 ± 0.060. The NPs’ content in the release medium was undetectable by DLS after 12 h of incubation.

The model that best fits the EVE release data was evaluated by correlation coefficients (R^2^). The higher correlation coefficient was revealed for the Higuchi model (R^2^ = 0.9690), demonstrating that, in this specific case, the EVE release from the HA/CS-TPP NPs’ matrix is diffusion-controlled. The R^2^ of 0.9228 and 0.8095 was calculated by fitting a zero-order and first order model, respectively. Finally, the regression coefficient and the release exponent (n) generated by fitting the EVE release data to the Korsmeyer–Peppas model equation were 0.9393 and 0.3531, respectively. These data further demonstrate that drug release from HA/CS-TPP NPs primarily occurs via diffusion [65,66].

### 2.4. “Ex Vivo” Cellular Tests

#### 2.4.1. Cytotoxicity

In previous works, it was stated that a high molecular weight HA regulates the cells cycle, suppressing their progression; moreover, the antimitogenic effect was observed in a different type of cell, including fibroblasts, and was correlated to the down regulation of the signal pathways of cyclin D [67,68].

**Figure 4:**
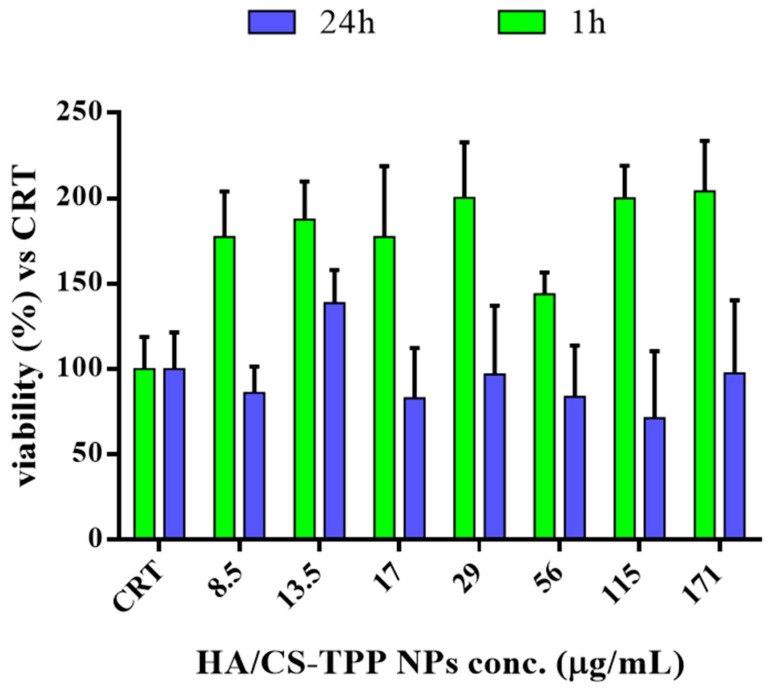
Cytotoxicity of placebo HA/CS-TPP NPs at 1st and 24th h of incubation with normal human dermal fibroblasts (NHDFs) compared to untreated control (CRT) cells.

The cytotoxicity study on the placebo HA/CS-TPP NPs was carried out on NHDFs, as a model cell line, in order to evaluate whether the HA and CS polymers have an effect on the in vitro cell proliferation. The cell viability was evaluated after 1 and 24 h of incubation by a microculture tetrazolium (MTT) assay. The results, plotted in Figure 4, were expressed as the cell viability percentage vs. the control (CRT). The CRT (cells incubated without NPs) was considered as 100% of the viability.

After the first hour of incubation, a noteworthy increase of the cell viability was detected in comparison with the control (CRT), because of the biocompatibility polymers’ properties and their stimulating action on cell proliferation, as fully reported in the literature [69,70].

After 24 h, the cell viability was always higher than 80%, the data are consistent with the optimal biocompatibility of the polymers, the excellent properties of the polysaccharides [41,54,71], and they are proof of a set-up preparation method of safety. Therefore, the HA/CS-TPP NPs can be considered safe candidates for drug delivery purposes. The poor effect of a high Mw HA on cell proliferation, as above mentioned, could be ascribed to the low concentration of HA moieties in the HA/CS-TPP NPs, with regards to the literature [68].

#### 2.4.2. Effect of EVE on NHDFs Growth

The effectiveness of the EVE loaded HA/CS-TPP NPs on the NHDFs growth was assessed by seeding the NPs re-suspended in Dulbecco’s modified Eagle’s medium (DMEM), with 10% (*v*/*v*) FBS and a 1% (*v*/*v*) penicillin/streptomycin solution with the NHDFs. Different concentrations of NPs were tested (8.5, 13.5, 17, 29, 56, 115, and 171 μg/mL) corresponding to 7.5, 10, 25, 50, 100, and 150 ng/mL of the EVE total amount into NPs, respectively. The EVE solutions, prepared as described in Section 3.6.3, were tested as the positive control. The NHDFs’ viability was evaluated by a MTT test after 1, 4, and 24 h of incubation, and was compared with the untreated cells (CRT).

**Figure 5:**
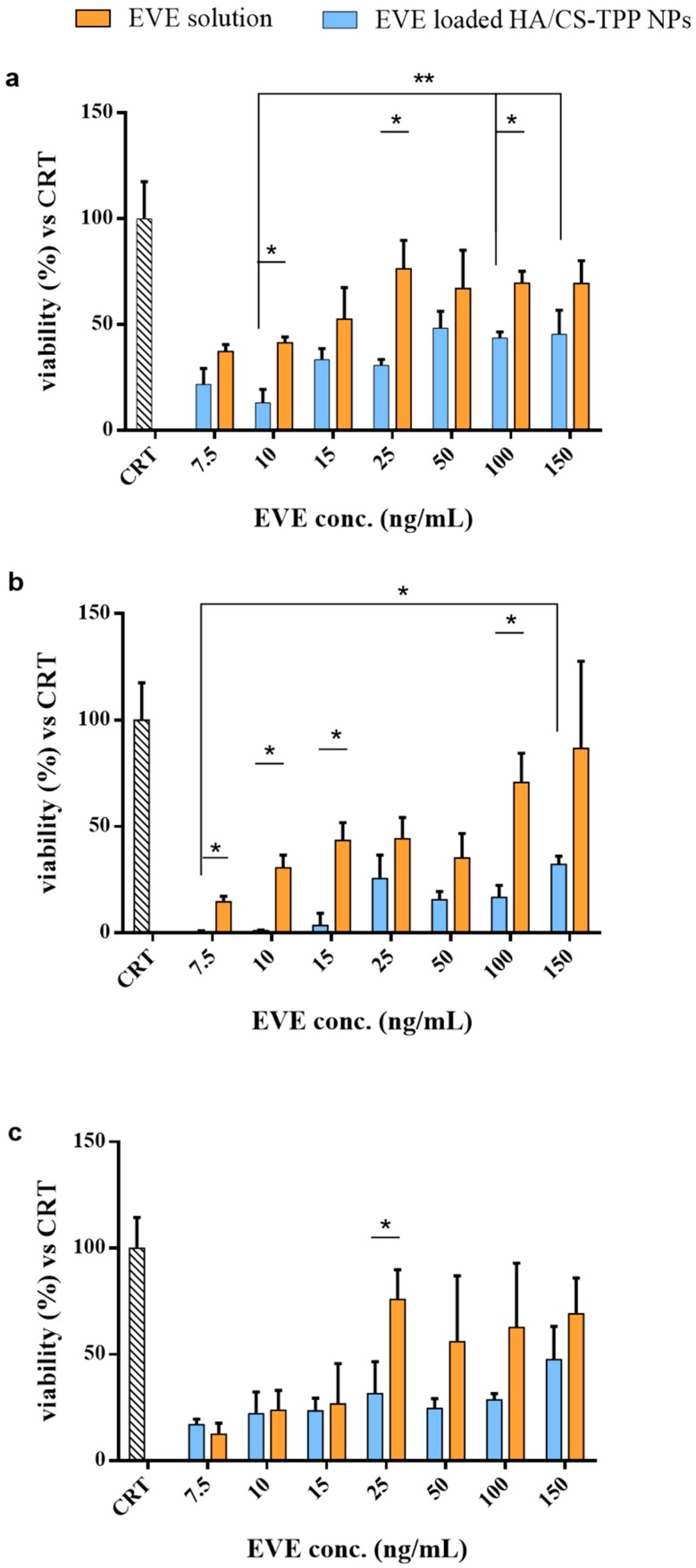
NHDFs’ viability after 1 h (**a**), 4 h (**b**), and 24 h (**c**) of incubation with EVE loaded HA/CS-TPP NPs (dark bar), EVE solutions (grey bar), and untreated control ([CRT], striped bar) cells. Statistical significance was determined using the Holm–Sidak method (multiple *t* test), with α < 0.05 (*). Tukey’s multiple comparisons test reveals a significant difference (*) for *p* values < 0.05 and (**) for *p* values < 0.01.

After 1 h of incubation (Figure 5a), the EVE loaded HA/CS-TPP NPs showed an inhibitory effect higher than the relative EVE solutions; the statistical significance was determined for the EVE concentration of 10, 25, and 100 ng/mL. The EVE loaded HA/CS-TPP NPs demonstrated a reduction in the cells’ growth, in a range of 45% to 80%, depending on the NPs’ amount, whereas the EVE solutions showed a maximum inhibitory effect of about 50%, regardless of its concentration.

Figure 5b presents the results collected after 4 h of incubation. A significant reduction of cell growth, ranging from 70% to 98%, was observed for all of the tested amounts of EVE loaded HA/CS-TPP NPs, revealing an anti-fibrotic efficiency substantially higher than the relative EVE solutions (20–80%).

These findings could be attributed to the NPs’ cell uptake rate and the release of the drug in the cytosol, which improves the EVE therapeutic efficacy. The EVE loaded HA/CS-TPP NPs’ efficiency increased after 4 h of incubation, depending on the drug release, and 30% of the EVE was released in the first h, following a rapid boost reaching 60% after 4 h.

The results collected after 24 h of incubation are shown in Figure 5c, excluding the EVE concentration of 25 ng/m; all of the concentrations tested did not underline the significant difference between the EVE loaded HA/CS-TPP NPs and the relative EVE solutions; in addition, the cell growth inhibition caused by the NPs was kept constant, ranging between 15–50%.

The highest inhibitory effects, regardless of the incubation time, were achieved with the lowest NPs’ concentrations. The trend demonstrates that the endocytosis process followed a saturable kinetic, due to the high binding affinity of the high molecular weight HA moieties for both the CD44 receptors [29,72,73] and the clathrin pits [42,56,74], ending in their saturation. Therefore, a specific number of NPs can pass the cell membrane and release the drug inside the cell, while the no internalized NPs release the EVE in external medium with any significant difference, compared with the EVE solution.

#### 2.4.3. Effect of EVE on NHDFs Proliferation

**Figure 6:**
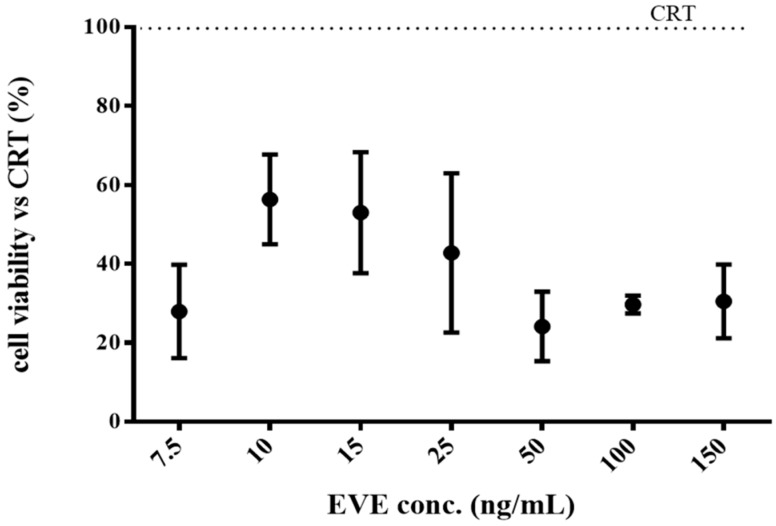
NHDFs proliferation: cells were incubated for 4 h with EVE loaded HA/CS-TPP NPs, then, the NPs were removed and the fresh Dulbecco’s modified Eagle’s medium (DMEM) was replaced. The MTT test was carried out after 24 h.

The inhibitory effect of the EVE loaded HA/CS-TPP NPs on the NHDFs’ proliferation was assayed, evaluating the reduction of the proliferation rate. The fibroblasts were incubated with EVE loaded HA/CS-TPP NPs for 4 h, then, the medium was removed and replaced with fresh DMEM. An MTT assay was performed after 24 h of incubation. Comparing the results in Figure 6 with the reduction of cell growth detected after 4 h of incubation (Figure 5b), it is clear that the cells slowly start to regrow; nevertheless, the proliferation rate is lower than the CRT sample, and the cell viability did not exceed 70%.

Moreover, the EVE loaded HA/CS-TPP NPs placed in contact with cells for 4 h caused a reduction in the proliferation cells of about 30% in the subsequent 24 h.

#### 2.4.4. Effect of EVE on MSc Growth

**Figure 7:**
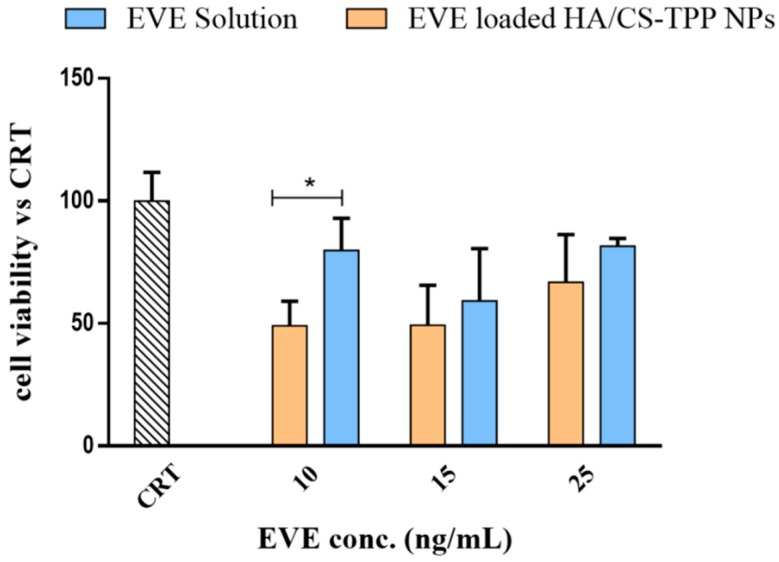
Mesenchymal cells’ (MSc) viability after 4 h of incubation with the EVE loaded HA/CS-TPP NPs and relative EVE solutions (untreated cells are indicated as CRT, striped bar). The statistical significance was determined using the Holm–Sidak method (multiple *t* test), with α < 0.05 (*).

As has already been stated by Cova et al., that the MSc isolated from the BOS-affected patients’ BAL can be considered as an ideal target as the ultimate BOS effector [17]. The effect of the EVE loaded HA/CS-TPP NPs’ on the MSc growth was evaluated by incubating the NPs with MSc at 37 °C in a 5% CO_2_ atmosphere for 4 h; the data have been compared to the results collected by the NHDFs. Different concentrations of the EVE loaded HA/CS-TPP NPs were tested, ranging between 13.5 and 29 μg/mL, corresponded to 1 and 25 ng/mL of the EVE. These concentrations have been selected from the best results that were previously obtained with NHDFs. The in vitro viability data of the MScs are shown in Figure 7.

The EVE loaded HA/CS-TPP NPs induced a significant negative effect on MSc growth, and the cell growth ranged from a 20% to 50% reduction, compared with the EVE solution, demonstrating a potential effectiveness in increasing the EVE antifibrotic activity.

As above mentioned, in the case of the NHDFs, the best result was achieved with the lowest NPs concentration, 13.5 μg/mL (corresponding to 10 ng/mL of EVE), for which multiple *t*-tests (Holm–Sidak method, α = 0.05) revealed a significant difference (*p* value = 0.001) between the NPs and the relative EVE solution. Nevertheless, the inhibitory effect of the EVE loaded HA/CS-TPP NPs on MSc was noticeably lower than that revealed on the NHDFs (70–98%). Postulating a receptor-mediated endocytosis uptake mechanism, the different behaviour of MSc and NHDFs could probably be due to the different CD44 isoforms expressed on the cell membranes [54] and to an overriding CD44-dipendent uptake mechanism in the CD44-overexpressing MSc.

#### 2.4.5. NPs Cellular Uptake Evaluation

Considering the difference of the EVE loaded HA/CS-TPP NPs’ inhibitory effect on the NHDFs and MSc from the BOS-affected patients’ BAL, the qualitative uptake studies were performed on fluorescently labelled HA/CS-FITC-TPP NPs.

To evaluate whether the HA decoration on the CS-TPP NPs has a statistically significant effect on the cellular uptake, the impact of the fluorescent HA/CS-FITC-TPP NPs were compared with the fluorescent CS-FITC-TPP NPs’ ones. The HA/CS-FITC-TPP NPs’ physical characteristics were consistent with the non-fluorescent HA/CS-TPP NPs showing particle sizes around 200 nm and a negative surface charge (−31.77 mV), while the CS-FITC-TPP NPs were synthetized with a mean size of 138.8 ± 86.2 nm, and a positive ζ potential (19.51 mV).

**Figure 8:**
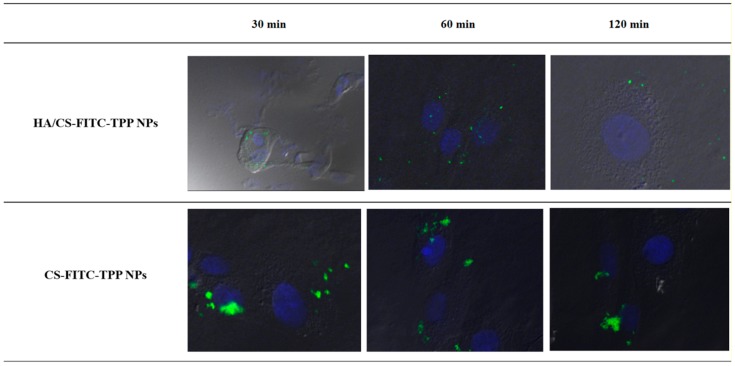
Confocal microscopy images of NHDFs (# 100,000 cells) incubated with HA/CS-FITC-TPP NPs and CS-FITC-TPP NPs at scheduled times (30, 60, and 120 min). Green excitation signals for HA/CS-FITC-TPP NPs and CS-FITC-TPP NPs, and blue excitation signals for cells nuclei. Magnification 63×.

Figure 8 shows the intracellular uptake behavior of the HA-decorated CS-FITC-TPP NPs and the CS-FITC-TPP NPs by NHDFs. The HA/CS-FITC-TPP NPs showed a slightly slow uptake; after 30 min of incubation, fluorescent NPs were found free in the cytosol; the highest internalized NPs’ amount was reached after 60 min of incubation; while no fluorescent NPs were detected inside the cells after only 120 min.

For the CS-FITC-TPP NPs, a small number of NPs were observed inside the NHDFs at 60 min (Figure 8). These findings were attributed to the evident NP aggregation phenomena, the aggregated NPs press on the phospholipid cell membrane, hampering their uptake into the cells. The aggregation in the culture media for the CS-TPP NPs is indicative of a relative in vivo colloidal instability; the ζ negative value of the NPs decorated with HA seem to provide high stability in cellular medium.

**Figure 9:**
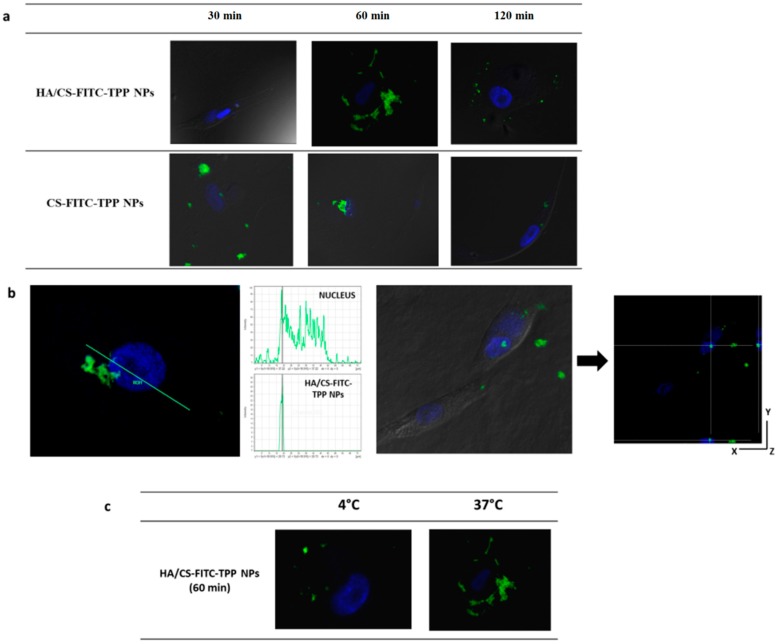
Confocal microscopy images of MSc (100,000) incubated with HA/CS-FITC-TPP NPs and CS-FITC-TPP NPs for scheduled times (30–60–120 min) (**a**); confocal optical section of MSc containing HA/CS-FITC-TPP NPs at 120 min of incubation. The internalization was confirmed by histogram analysis of the fluorescence intensities along the yellow line. The image shows the cross sections in the XZ- and YZ-axes as the cell height and width, respectively (**b**); HA/CS-FITC-TPP NPs inside cells after 120 min of incubation at 4 °C and 37 °C (**c**). Green excitation signals for HA/CS-FITC-TPP NPs and CS-FITC-TPP NPs, and blue excitation signals for cells nuclei. Magnification 63×.

In Figure 9, the intracellular uptake performances of the HA/CS-FITC-TPP NPs and CS-FITC-TPP NPs cultured with MSc are illustrated. Figure 9a shows that the HA decoration on the NPs dramatically affected the kinetic and the yield of NPs uptake in MSc slowing down the uptake rate induced by a more specific binding to the receptors (possibly CD44) with respect to CS-FITC-TPP NPs. The HA/CS-FITC-TPP NPs were clearly identified on the cells surface, forming a shell around the cells at 60 min of incubation. This can be attributed to the strong interaction of the high molecular HA chains with the receptor increasing the avidity as a result of multiple binding sites on each HA moiety. At 120 min of incubation, the NPs were partially co-located near the phospholipid membrane, most likely caused by re-binding events during the HA dissociation from the receptor and a small amount of HA/CS-FITC-TPP NPs accumulated inside the cell cytoplasm, as better highlight in Figure 9b, where the co-localization of the HA/CS-TPP NPs and nucleus signals were confirmed by the histogram analysis of fluorescence intensities along the yellow line.

The cytoplasmic distribution at 120 min of incubation was also verified by a z-axis transformation analysis with optical zoom, the cell nucleus was used as internal reference point, and the XZ and YZ planes show the cell height and width; HA/CS-FITC-TPP NPs were localized in perinuclear region (Figure 9b). These results show the same trend observed by Mizrahy et al. [72] for the free HA molecules. The CS-TPP-FITC NPs revealed a faster uptake (within 30 min), and the NPs’ intracellular level remained stable until 60 min of incubation, while at 120 min, only trace amounts of fluorescent NPs were present inside the cells, mostly because of the non-specific, clathrin-mediated uptake mechanism.

The NPs’ cellular uptake and cellular localization were confirmed to be cell-line dependent, focusing on MSc, the NPs’ surface composition determines the kinetic of the internalization controlling the binding of the NPs on the cell surface.

In order to better confirm the CD44-mediated cellular uptake process, the HA/CS-FITC-TPP NPs were incubated with MSc at 4 °C, which is the temperature at which the receptor activity is substantially inhibited. The behavior at 4 °C was dramatically different with respect to the data collected at 37 °C, showing a poor uptake and confirming the involvement of an active NP’s uptake mechanism, attributed to the CD44 regulation (Figure 9c).

**Figure 10:**
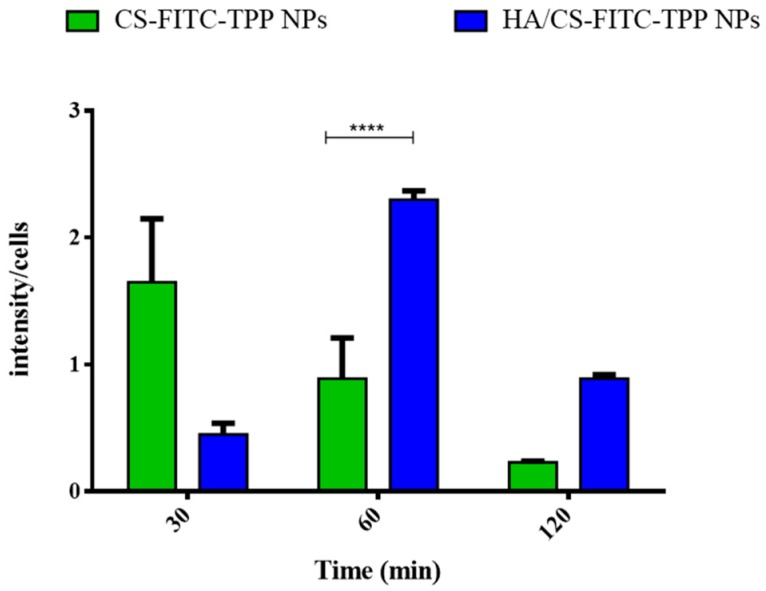
Quantitative evaluation of the cellular uptake studies performed on MSc from the broncoalveolar lavage of the bronchiolitis obliterans syndrome (BOS)-affected patients with HA/CS-FITC-TPP NPs at 37 °C. CS-FITC-TPP NPs were used as the control. The results are expressed as fluorescence intensity/cell vs. time, as showed in the histogram. The data represent the mean ± SD (*n* = 3). Statistical significance was determined using the Holm–Sidak method (multiple *t* test), with α < 0.05 (*). Tukey’s multiple comparisons test reveals a significant difference (****) for *p* values < 0.0001.

A quantitative evaluation of the NPs’ cellular uptake was performed by an image analysis software tool [75,76]. The results, expressed as fluorescence intensity/cell as a function of time (Figure 10), show that both the CS-FITC-TPP and HA decorated CS-FITC-TPP NPs were uptaken by CD44-overexpressing MSc from BOS-affected patients’ BAL. The main difference can be attributed to the uptake profile that is higher for the HA/CS-FITC-TPP NPs, starting from 60 min of incubation, and more prolonged until 120 min, with respect to what was noticed for the un-targeted CS-FITC-TPP NPs. This behavior can be useful to fine target and prolong the therapeutic effect of the loaded active agents.

## 3. Materials and Methods

### 3.1. Materials

The chitosan chloride (CS), of pharmaceutical grade, (PROTASAN CL 113, Mw 110 kDa, deacetylation degree 75–90%, Chloride content 10–20%) was purchased from FMC BioPolymer AS NovaMatrix, (Oslo, Norway). The hyaluronic acid sodium salt (HA), from Streptococcus equi, (high molecular weight, Mw 1500 kDa), Sodium tripolyphosphate ((TPP), Mw 367.86 g/mol), Cetyltrimethylammonium bromide, 85% (CTAB, Mw 378.49), Everolimus of an analytical standard (EVE, Mw 958.22 g/mol), Thiazolyl Blue Tetrazolium Bromide (MTT, approx. 98% TLC), and Cibacron Brilliant Red 3B-A (dye content 50%, Mw 995.23 g/mol) were obtained from Sigma Aldrich (St. Louis, MO, USA); and Sodium chloride (NaCl, Mw 58.443 g/mol), Sodium phosphate monobasic (NaH_2_PO_4_, Mw 119.98 g/mol), and Potassium phosphate monobasic (KH_2_PO_4_, Mw 136.09 g/mol) were purchased from Carlo Erba Reagents (Cornaredo, Milano, Italy). The Dulbecco’s modified Eagle’s medium ((DMEM), with glucose 4.5 g/L and l-glutamine) was from BioWhittaker (Lonza, Verviers, Belgium). The water used in the preparation of polymeric solutions was distilled and filtered through 0.22 m membrane filters (Millipore Corporation, Billerica, MA, USA).

The normal human dermal fibroblasts’ (NHDFs) adult skin was purchased from PromoCell (VWR International PBI s.r.l., Milano, Italy); the mesenchymal cells (MSc) were isolated from the bronchoalveolar lavage (BAL) of BOS-affected patients, following the standard technical recommendation reported by the European Society of Pneumology Task Group (1989).

Unless specified, all of the other solvents and reagents were of analytical grade.

### 3.2. HA/CS-TPP Nanoparticles Preparation Method Set-Up

A one-step IG method was developed and named the inverse ionotropic gelation (IIG) method, in order to highlight both the opposite dropping methodology of the polymeric phases, with respect to the most conventional IG nanoprecipitation method and the negative ζ potential of NPs obtained in all of the process conditions. Briefly, 4 mL of CS aqueous solution (0.1125–1 mg/mL) was added dropwise at constant flow rate of 1 mL/min using 27 G needle into an aqueous solution containing 4 mL of HA solution (0.5–2 mg/mL) and 50 µL of TPP solution (0.25–0.5 mg/mL); the system was maintained under magnetic stirring (700 rpm) at room temperature during the whole dropping phase. Magnetic stirring was maintained for 10 min to allow for the stabilization of the system. The NPs’ suspension was subsequently transferred to the centrifuge tubes and the NPs were recovered by centrifugation at 16,400 rpm for 10 min at 4 °C in 60 µL of a glycerol bed, to prevent NPs aggregation.

IIG was set up by an experimental design procedure (DoE, see Section 3.2.1), considering the CS concentration, HA concentration, and TPP concentration as critical process variables, in order to produce NPs with physico–chemical properties suitable for the pulmonary application (size < 200 nm, narrow size distribution and negative ζ potential).

The process yield was gravimetrically evaluated on NP batches freeze-dried at −50 °C, 0.01 bar for 24 h (Lio 5P, Cinquepascal s.r.l., Milano, Italy), and expressed as a % ratio between the total weight of the NPs’ batch after recovering, and the weight of the raw materials (HA, CS, and TPP) used for each NPs batch.

#### 3.2.1. Screening Design of Experiment (DoE)

A screening design of experiment (DoE) was performed (Statgraphics Centurion XVII) to characterize and establish the design space by identifying critical aspects (such as the HA, CS, and TPP concentration) crucial for the product characteristics (NPs’ size and ζ potential). A full factorial design (2^n^) was set up in order to identify the process variables (input) with a significant effect on the responses (output). The CS, HA, and TPP concentrations were selected as the main inputs and their effect on the particle size and ζ potential was evaluated. In this design for a 2^n^ full factorial design, the number of possible combinations between different inputs is 2^3^, where 2 is the number of levels tested and n (3) is the number of inputs studied. For each input, a minimum and maximum level were defined (*−1* and +*1* respectively) and a central point, which is the mean value (*0*). Table 2 reports the levels of the CS, HA, and TPP concentration tested.

### 3.3. Nanoparticles Characterization

#### 3.3.1. Nanoparticles Dimensional and Morphological Characterization

The volume-average particle diameter of the blank and EVE loaded HA/CS-TPP NPs was assessed using dynamic light scattering (DLS) NICOMP 380 ZLS apparatus (Particle Sizing Systems, Menlo Park, CA, USA). The polydispersity index (PDI) was also evaluated in order to describe the particle size distribution; a PDI value of about 0.3 is considered the maximum limit for a monodisperse polysaccharides-based NPs population [77]. The sample run time was approximately 15 min.

For the ζ potential measurements, the samples were diluted with a 10 mM KCl aqueous solution, at run time of 60 s. All of the measurements were made in triplicate, and the mean values ± standard deviation (SD) were recorded.

Transmission electron microscopy (TEM) (TEM-208S, Philips, Eindhoven, The Netherlands) was used for imaging the plain and EVE loaded HA/CS-TPP NPs. Then, 15 μL of HA/CS-TPP NPs’ aqueous suspension were stained with 1% (*w*/*v*) phosphotungstenic acid at pH 7 (adjusted with NaOH solution, 0.1 M) for 2 min, and then immobilized on copper grids to be viewed by TEM.

The morphological examination of the selected plain and EVE loaded HA/CS-TPP NPs was executed using scanning electon microscopy (SEM) (Zeiss EVO MA10 (Carl Zeiss, Oberkochen, Germany), using the gold sputter technique. The samples were prepared by drying a drop of diluted NPs suspension on a sample stub at 37 °C overnight. Afterwards, the samples were sputtered with a gold layer (Leica EM SCD 500, Leica Microsystems GmbH, Wetzlar, Germany), and then the microscopic observation was performed at an accelerating voltage of 20 kV and a working distance of 8.5 mm.

#### 3.3.2. HA/CS-TPP Nanoparticles Polymeric Composition

##### Hyaluronic Acid Quantification

A liquid chromatography method (HPLC) method based on the size exclusion liquid chromatography with UV detection was used to quantify the HA in the NPs’ supernatant, after recovering by centrifuge at 16,400 rpm for 20 min. The method was developed and validated by Ruckmani et al. [78]. The analyses were carried out on a Yarra SEC2000 300 mm × 7.8 mm column, with 3 μm silica particle size and 145 Å pore size at 25 °C. Potassium dihydrogen phosphate (pH 7) was used as isocratic mobile phase at a flow rate of 1 mL/min and a detection wavelength was set up at 205 nm. The chromatographic experiments were performed on Agilent 1260 infinity HPLC (Agilent Technologies, Milan, Italy), with an isocratic pump (1260 Iso Pump) and UV detector (Agilent 1260 Series UV-visible detectors), and a manual injector (Agilent 1260 infinity manual injector, injection volume 20 μL). The calibration curve was obtained by linear regression in the 0.3–0.5 mg/mL HA concentration range, with an acceptable correlation coefficient of 0.9885.

The HA percentage (% HA) effectively reacts with CS during the NPs’ formation, which was calculated as follows:

% HA = 100 × (HA_pol_ − HA_sup_)/HA_pol_ (3)

where HA_pol_ is the amount of polymer used in the preparation of each NPs batch, and HA_sup_ was the free HA amount, spectrophotometrically detected, into supernatant volume. Afterwards, the HA percentage of the HA/CS-TPP NPs’ batch was effectively (% HA_NPs_) calculated, as follows:

% HA_NPs_ = 100 × (HA_pol_ − HA_sup_)/*W*_b_ (4)

where *W*_b_ is the total weight of each NPs batch after freeze-drying (see Section 3.2).

##### Chitosan Quantification

A Cibacron Brilliant Red 3B-A colorimetric assay [79,80,81] was used to evaluate the amount of CS reacts forming a complex with TPP and/or HA in the NPs formation. The supernatants (100 μL) were collected by centrifuge and diluted into a mixture containing 200 μL of glycine buffer and 3 mL of a dye solution. A final sample was analyzed using a UV-VIS spectrophotometer (6705 UV-vis spectrophotometer, JENWAY, Staffordshire, UK) at 575 nm. A reference solution consisted of a solution containing a glycine buffer (300 μL) and dye solution (3 mL).

The chitosan association efficiency (% CS) was determined, above described as Equation (5), as follows:

% CS = 100 × (CS_pol_ − CS_sup_)/CS_pol_ (5)

where CS_pol_ was the amount of polymer contained in 4 mL of the CS solution used in the preparation of each NPs batch, and CS_sup_ was the free CS amount, spectrophotometrically measured, present in each supernatant volume.

The composition of each HA/CS-TPP NPs batch (% CS_NPs_) was calculated as follows:

% CS_NPs_ = 100 × (CS_pol_ − CS_sup_)/*W*_b_ (6)

where *W*_b_ was the total weight of each NPs batch recovered after freeze-drying (see Section 3.2).

##### Fourier Transform Infrared (FTIR) Spectroscopy

The FTIR spectra of the placebo HA/CS-TPP NPs, previously freeze-dried at −50 °C, 0.01 bar for 24 h (Lio 5P, Cinquepascal s.r.l., Milano, Italy), and the raw polymers (CS and HA), were recorded using a Thermo Scientific Nicolet iN10 spectrometer (Waltham, MA, USA). A sample analysis was performed using the ATR mode with a Ge crystal. The spectra were obtained at 256 scans and a resolution of 2 cm^–1^.

### 3.4. EVE Loaded HA/CS-TPP Nanoparticles Preparation

The set-up IIG method was exploited for preparing the EVE loaded HA/CS-TPP NPs. Briefly, 200 μL of the EVE stock solution (0.1 mg/mL in a methanol:water mixture, 1:1 *v*/*v*) was added to the CS solution, to be poured into the HA/TPP solution, as described above (see Section 3.2).

### 3.5. Drug Loading and In Vitro Release of EVE Loaded HA/CS-TPP Nanoparticles

An HPLC method was in house-modified to quantify the amount of EVE encapsulated in NPs, and to study the release profile [82,83]. The analyses were carried out on a Zorbax Eclipse^®^ Plus C18 Chromatography Column, 4.6 mm × 150 mm, 5 μm heated at 55 °C. The mobile phase was prepared by mixing purified water, methanol, and acetonitrile (ratio 22:60:18); the flow rate was fixed at 1.5 mL/min; and the detection wavelength was fixed at 278 nm. All of the chromatographic experiments were carried out on an Agilent 1260 HPLC (Agilent Technologies, Milan, Italy) consisting of a pump (1260 Infinity Quaternary Pump VL), UV detector (Agilent 1260 Series UV-visible detectors, multi-wavelength detector), and manual injector (Agilent 1260 Infinity Manual Injector). The calibration curve, in a concentration range from 1 to 10 μg/mL, had a linearity of R^2^ = 0.9969, all of the measurements were ranged in the level of detection (LOD 0.30 μg/mL) and level of quantification (LOQ 1 μg/mL). The HPLC method showed a recovery of 98.4 ± 1.7%.

#### 3.5.1. EVE Loading

The supernatants recovered by centrifuge were diluted 1:1 (*v*/*v*) with methanol to prevent the EVE precipitation, and were analyzed by HPLC. The EVE loaded into the NPs was expressed as μg of EVE/100 mg of NPs, and was calculated as follows:

EVE loading = 100 × (EVE_w_ − EVE_sup_)/*W*_batch_ (7)

where EVE_w_ are micrograms of the EVE used for the preparation of each NPs batch, EVE_sup_ are micrograms of the EVE detected into the supernatant, and *W*_batch_ is the weight (mg) of NPs recovered after freeze-drying.

#### 3.5.2. In Vitro EVE Release

Taking into consideration the EVE hydrophobic feature, and in order to perform an in vitro release test in sink the conditions, a minimum amount of Tween 20 was added to the release medium and the test was performed, as below described.

Firstly, 6 mg of fresh NPs, theoretically containing 5.8 μg of EVE, were suspended in 1 mL of PBS (pH 7.4), supplemented with 0.4% (*w*/*v*) Tween 20; then, a release test was performed at 37 °C under tilting agitation. At schedule times (30 min, 1, 2, 4, 6 and 24 h), samples were centrifuged (16,400 rpm) for 30 min at 25 °C, and 500 μL of supernatants were collected and replaced with a fresh buffer. The supernatant was diluted with 250 μL of methanol and was analysed by HPLC, as above described. The EVE release data were plotted as (i) a cumulative amount of the drug released versus the time (zero order kinetics model); (ii) ln cumulative percentage of the drug released versus the time (first-order kinetics model); and (iii) a cumulative percentage of the drug release versus the square root of time (Higuchi kinetic model). Furthermore, the EVE release data until 60% were processed by the empirical Ritger and Peppas equation [66].

### 3.6. Ex Vivo Cellular Tests

#### 3.6.1. Cells

The NHDFs and MSc from the BOS-affected patients’ BAL were separately cultured in DMEM supplemented with 10% (*v*/*v*) FBS and 1% (*v*/*v*) penicillin/streptomycin solution. The cells were trypsinized when subconfluent and were seeded in a 96-well multiwell plate at 37 °C in a 5% CO_2_ atmosphere (10,000 cells per well). The MSc surface phenotypes were previously characterized, confirming CD44 overexpression [17].

#### 3.6.2. HA/CS-TPP Nanoparticles Cytotoxicity

The preliminary cytotoxicity tests were performed incubating the NHDFs with placebo HA/CS-TPP NPs for 1 and 24 h at 37 °C in a 5% CO_2_ atmosphere. The NPs recovered after centrifugation were re-suspended in sterile DMEM, with a 10% (*v*/*v*) FBS and 1% (*v*/*v*) penicillin/streptomycin solution. Different concentrations of NPs were tested (8.5, 13.5, 17, 29, 56, 115, and 171 μg/mL), and the cell viability was evaluated using the MTT assay [84].

The results were read on a multiwell scanning spectrophotometer (Microplate Reader Model 680, Bio-Rad Laboratories, Segrate, Italy). The absorbance was measured at 570 nm, with 690 nm as a reference wavelength. The cell viability was calculated as a percentage of the untreated cells (control).

#### 3.6.3. Effect of EVE Loaded HA/CS-TPP Nanoparticles on NHDFs Growth and Proliferation

The EVE loaded HA/CS-TPP NPs were incubated with NHDFs at 37 °C in a 5% CO_2_ atmosphere for 1, 4, and 24 h, to estimate the immediate anti-fibrotic effect of the EVE released from the HA/CS-TPP NPs. The NPs recovered by centrifuge were re-suspended in sterile DMEM, with a 10% (*v*/*v*) FBS and 1% (*v*/*v*) penicillin/streptomycin solution, and different concentrations of NPs were tested (8.5, 13.5, 17, 29, 56, 115, and 171 μg/mL), corresponding to 7.5, 10, 25, 50, 100, and 150 ng/mL of EVE into the NPs. The cell viability was assessed by MTT assay, as previously described.

To evaluate the inhibitory effect on cell proliferation, the test was performed on the EVE loaded HA/CS-TPP NPs (8.5, 13.5, 17, 29, 56, 115, and 171 μg/mL) incubated with NHDFs for 4 h; following DMEM with NPs, the suspension was removed and fresh DMEM was re-placed. The MTT test was carried out, as previously described, after 24 h of incubation.

The NHDFs’ viability was also detected using different EVE solutions (7.5, 10, 25, 50, 100, and 150 ng/mL) and used as a reference; the solutions were prepared by the dilution of the EVE stock solution (0.1 mg/mL) with sterile DMEM, with a 10% (*v*/*v*) FBS and 1% (*v*/*v*) penicillin/streptomycin solution.

#### 3.6.4. Effect of EVE Loaded HA/CS-TPP Nanoparticles on MSc Growth

The EVE loaded HA/CS-TPP NPs were incubated with MSc from the BOS-affected patients’ BAL, at 37 °C in a 5% CO_2_ atmosphere for 4 h.

The NPs recovered after centrifugation were re-suspended in sterile DMEM, with a 10% (*v*/*v*) FBS and 1% (*v*/*v*) penicillin/streptomycin solution; different concentrations of NPs were tested (13.5, 17 and 29 μg/mL) corresponding to 10, 25, and 50 ng/mL of the EVE total amount into the NPs. The cell viability was assessed using the MTT assay, as previously described.

Then, 10, 25, and 50 ng/mL EVE solutions were prepared, as reported above (see Section 3.6.3), and were used as reference.

#### 3.6.5. Nanoparticles Cellular Uptake Determination

A confocal microscopy was used to assess the HA/CS-TPP NPs uptake by NHDFs and MSc cultured from the BOS-affected patients’ BAL. The fluorescent NPs (HA/CS-FITC-TPP NPs) were prepared using FITC-labelled CS (CS-FITC) [55,85], according to the previously set-up IIG method.

Fluorescent CS based NPs (CS-FITC-TPP NPs), prepared by the IG method previously set-up in our laboratory [56] and characterized for size and ζ potential, were used as the control (uncoated NPs).

To determine whether the NPs’ cellular uptake was receptor-mediated, the NPs recovered by centrifuge were re-suspended (200 g/mL) in sterile DMEM, with 10% (*v*/*v*) FBS and a 1% (*v*/*v*) penicillin/streptomycin solution. Then, 2 mL of HA/CS-FITC-TPP NPs or CS-FITC-TPP NPs suspension were incubated with the MSc previously seeded on two 12 mm glass bottom slides per well (100,000 cells for each slide), and incubated at 37 °C and 4 °C. At scheduled times (30, 60, and 120 min), the glass slides were washed with sterile 1× PBS and fixed with 4% (*w*/*v*) paraformaldehyde in a PBS buffer, and incubated for 10 min at 4 °C. The short times of incubation were chosen because longer incubation times at 4 °C caused a drastic decrease in the cell viability, also in the absence of NPs.

The cells were rinsed with PBS and fixed for the observation cell; the cell nuclei were counterstained with a DAPI solution in PBS (800 nM). The specimens were examined under a confocal laser scanning microscope (Leica TCS SP2, Leica Instruments, Nussloch, Germany), and the spaced optical sections were recorded using a 63× oil immersion objective for each cells line; each uptake experiment was performed in triplicate and three images of each time point were analysed.

The uptake amount was quantified on the samples incubated at 37 °C by measuring the NPs’ fluorescence inside the cells using ImageJ software. The experiment was performed in triplicate. Three different images were collected for each time point, each representing 20 cells at least. The results are expressed as fluorescence intensity/cell. The fluorescently labelled untargeted NPs (CS-FITC-TPP NPs) were used as the control.

##### CS-FITC Synthesis

Briefly, 50 mg of CS was dissolved in 5 mL of 0.1 M acetic acid, obtaining a polymer solution of 1% (*w*/*v*), in which 5 mL of methanol was gradually added under magnetic stirring. The CS-FITC was synthetized adding 2.5 mL of FITC in methanol (2 mg/mL); the reaction was run for 3 h in the dark and at room temperature.

Afterwards, the CS-FITC was precipitated in a 0.5 M NaOH aqueous solution (till to pH 10). The precipitate was recovered by centrifugation at 16,400 rpm, at 4 °C for 10 min and washed in methanol: water (70:30 *v*/*v*). The washing and the pelletization procedures were repeated until no fluorescence was detected in the supernatant (Lumiscence Spectrophotometer LS55, Perkin Elmer, Whaltham, MA, USA). The CS-FITC was then dissolved in 0.1 N of acetic acid and dialyzed in the dark against distilled water for three days. Finally, the CS-FITC was freeze-dried [56].

The labelling efficiency was determined by measuring the florescence intensity of the CS-FITC solution against the FITC standard solution (Lumiscence Spectrophotometer LS55, Perkin Elmer, Whaltham, MA, USA) (λ_ex_ = 460 nm, λ_em_ = 517 nm; 28,154 calibration curve slope and 406.82 intercept with a good correlation coefficient (R^2^) of 0.9942 given in the 0.1–1 μM concentration range). The degree of labelling was calculated about 76.6 μg FITC/g CS.

### 3.7. Statistical Analysis

The statistical significance of the differences was determined by application of a two-way analysis of variance (ANOVA) with a Tukey multiple comparison test and a multiple *t*-test (Holm-Sidak method).

The differences were considered significant at *p* value < 0.05. All of the statistical analyses were performed in GraphPad Prism version 5 (GraphPad Software Inc., La Jolla, CA, USA).

## 4. Conclusions

A mild, one-step IIG method was successfully set-up by a DoE approach to formulate highly biocompatible and biodegradable HA-decorated CS-TPP NPs based on 60% CS and 40% HA, with a mean diameter of about 200 nm, good PDI (<0.2), and negative surface charge of about −30 mV, demonstrating a robust surface HA decoration. Moreover, the one-step IIG method from the handstand point of manufacturing was found simple, reproducible, and easy to scale up, and was likely to result in a product with targeted internalization.

A growth test along with the uptake studies performed on the MSc from the BOS-affected patients’ BAL highlighted that the HA decoration on the EVE loaded HA/CS-TPP NPs surface could boost their preferential internalization in the cells’ overexpressing receptors for HA (such as CD44), inducing a more effective effect of EVE in reducing the cells’ viability compared with the EVE solutions. The cell viability reduction ranged from 20% to 50% after 4 h of incubation and it was ascribable to the NPs’ uptake mechanism.

The good EVE loading into HA/CS-TPP NPs and its modulated in vitro release by 24 h could open new perspectives for a safe, pulmonary clinical therapy for chronic lung allograft dysfunction.

## Figures and Tables

**Table 1:** Hyaluronic acid (HA), chitosan (CS), and pentasodium tripolyphosphate (TPP) solution concentrations (input variables) and HA/CS-TPP nanoparticles (NPs) sizes and ζ potentials (output variables) used in the design of experiment (DoE) approach. SD—standard deviation; PDI—polydispersity index.

DoE Batch #	HA Solution (mg/mL) *(Level)*	CS Solution (mg/mL) *(Level)*	TPP Solution (mg/mL) *(Level)*	Size ± SD (nm)	PDI ± SD	ζ Potential ± SD (mV)
A	1 *(−1)*	0.3 *(−1)*	0.25 *(−1)*	385.2 ± 90.7	0.240 ± 0.096	−1.1 ± 0.3
B	1 *(−1)*	0.5 *(+1)*	0.5 *(+1)*	181.9 ± 65.6	0.171 ± 0.040	−30.9 ± 2.5
C	1 *(−1)*	0.5 *(+1)*	0.25 *(−1)*	195.4 ± 75.2	0.421 ± 0.163	−1.5 ± 0.8
D	1 *(−1)*	0.3 *(−1)*	0.5 *(+1)*	210.8 ± 94.9	0.202 ± 0.086	−10.8 ± 2.8
E	2 *(+1)*	0.5 *(+1)*	0.5 *(+1)*	242.5 ± 197.9	0.334 ± 0.103	−38.5 ± 5.2
F	2 *(+1)*	0.3 *(−1)*	0.5 *(+1)*	320.9 ± 322.4	0.382 ± 0.087	−40.5 ± 8.3
G	2 *(+1)*	0.3 *(−1)*	0.25 *(−1)*	630.5 ± 343.4	0.275 ± 0.069	−25.5 ± 4.6
H	2 *(+1)*	0.5 *(+1)*	0.25 *(−1)*	310.8 ± 162.2	0.272 ±0.023	−18.7 ± 3.9
I	1.5 *(0)*	0.4 *(0)*	0.375 *(0)*	230.3 ± 144.6	0.393 ± 0.042	−13.5 ± 1.6
L	1.5 *(0)*	0.4 *(0)*	0.375 *(0)*	223.1 ± 114.7	0.264 ± 0.062	−12.8 ± 3.2

**Table 2:** Selected inputs and relative levels for the screening DoE.

Input	*Level*		
	*−1*	*0*	*+1*
HA solution (mg/mL)	1	1.5	2
CS solution (mg/mL)	0.3	0.4	0.5
TPP solution (mg/mL)	0.25	0.375	0.5
